# A web-based prognostic tool for extremity and trunk wall soft tissue sarcomas and its external validation

**DOI:** 10.1038/bjc.2012.48

**Published:** 2012-02-21

**Authors:** M Sampo, M Tarkkanen, E Tukiainen, P Popov, P Gustafson, M Lundin, T Böhling, C Blomqvist, J Lundin

**Affiliations:** 1 Department of Pathology, HUSLAB and University of Helsinki, PO Box 400, HUCH-00029, Helsinki, Finland; 2 Department of Oncology, Helsinki University Central Hospital (HUCH), PO Box 180, HUCH-00029, Helsinki, Finland; 3 Department of Plastic Surgery, HUCH, PO Box 288, HUCH-00029, Helsinki, Finland; 4 Department of Orthopedics, Lund University, Sweden, Lund University Hospital, SE-221 85 Lund, Sweden; 5 Institute for Molecular Medicine Finland, University of Helsinki, PO Box 20, 00014, Helsinki, Finland; 6 University Division of Global Health, Karolinska Institutet, Stockholm, Sweden

**Keywords:** soft tissue sarcoma, prognosis, web-based, chemotherapy

## Abstract

**Background:**

We developed a web-based, prognostic tool for extremity and trunk wall soft tissue sarcoma to predict 10-year sarcoma-specific survival. External validation was performed.

**Methods:**

Patients referred during 1987–2002 to Helsinki University Central Hospital are included. External validation was obtained from the Lund University Hospital register. Cox proportional hazards models were fitted with the Helsinki data. The previously described model (SIN) includes size, necrosis, and vascular invasion. The extended model (SAM) includes the SIN factors and in addition depth, location, grade, and size on a continuous scale. Models were statistically compared according to accuracy (area under the ROC curve=AUC) of 10-year sarcoma-specific survival prediction.

**Results:**

The AUC of the SAM model in10-year survival prediction in the Helsinki patient series was 0.81 as compared with 0.74 for the SIN model (*P*=0.0007). The corresponding AUCs in the external validation series were 0.77 for the SAM model and 0.73 for the SIN model (*P*=0.03). A web-based calculator for the SAM model is available at http://www.prognomics.org/sam.

**Conclusion:**

Addition of grade, depth, and location as well as tumour size on a continuous scale significantly improved the accuracy of the prognostic model when compared with a model that includes only size, necrosis, and vascular invasion.

Soft tissue sarcomas (STS) are rare malignant neoplasms comprising approximately 1% of all cancers. Despite optimal local treatment and satisfactory local control rates, the percentage of STS patients developing metastases can be 20–30% (Potter *et al*, 1985; Huth and Eilber, 1988; Billingsley *et al*, 1999). Most patients dying of STS have metastases.

In a recent meta-analysis, adjuvant anthracycline-based combination chemotherapy significantly improved both relapse-free and overall survival in STS (Pervaiz *et al*, 2008). The combination of anthracyclines and ifosfamide seemed to be most efficacious, yielding a relative risk reduction of 0.56 and an absolute risk reduction of 11% for mortality. This moderate risk reduction has to be weighed against toxicity. The meta-analysis has been criticised for including many small studies, the oldest ones dating back to 1981. The largest randomised study failed to show a survival advantage for adjuvant chemotherapy (doxorubicin-ifosfamide) among patients with high-grade STS. So far, only meeting abstract has been published (Woll *et al*, 2007). In an adjuvant randomised phase II study by the Canadian sarcoma group, the combination of doxorubicin (50 mg m^−2^ per cycle) and ifosfamide (5 g m^−2^ per cycle) was associated with a 29% incidence of grade 3–4 nausea and vomiting and an 8% incidence of grade 3–4 haematological toxicity (Gortzak *et al*, 2001). Of the 67 patients (1.5%) died of neutropenic infection. Selection of patients to receive adjuvant chemotherapy is therefore critical. Practical tools for decision-making are scarce.

Gustafson *et al* (2003) constructed a new prognostic tool, the SIN system (size, vascular invasion, necrosis) in which the three adverse factors were tumour size greater than 8 cm, presence of vascular invasion, and tumour necrosis of any size. Of the 200 patients, 80 patients (40%) had two or three of the three adverse factors with metastases-free survival (MFS) of 43% at 5 years and 120 patients (60%) had none or only one adverse factor with MFS of 83% at 5 years (*P*<0.0001). Later, peripheral tumour growth pattern (infiltrating *vs* pushing growth) was added to the SIN model to predict metastases, and patients with vascular invasion and at least two other risk factors were high-risk patients showing specificity of 85% and positive predictive value of 65% (Carneiro *et al*, 2011).

Kattan *et al* (2002) used a database of 2136 patients to construct a prognostic tool and a nomogram for 12-year sarcoma-specific death on the basis of Cox regression model with a concordance index of 0.77. The model includes age, size, grade, histological subtype, depth, and site. It was later externally validated at the University of California Los Angeles (Eilber *et al*, 2004) and at Istituto Nazionale per lo Studio e la Cura dei Tumori, Milan, Italy (Mariani *et al*, 2005).

In 1987, a multidisciplinary STS group began a prospective treatment protocol at Helsinki University Central Hospital. Amendment to protocol was added in 1998 concerning adjuvant chemotherapy: patients less than 70 years and with sufficient performance status are offered adjuvant doxorubicin-based chemotherapy based on the previously mentioned SIN prognostic index: tumour malignancy grade is high (III–IV in a four-tiered scale) and the tumour fulfills at least two of the following criteria: size greater than 8 cm (in synovial sarcomas >5 cm), necrosis, or vascular invasion. The aim of the current study was to assess if the prognostic accuracy of a model with size, necrosis, and vascular invasion can be improved by addition of tumour depth, location, and histological grade. A secondary objective was to make the final model as a prognostic tool for the commonest histological subtypes of the extremity and the trunk wall STS with no targeted therapy available online for scientific use and future validations for the time being. External validation was performed on STS data obtained from the Lund University Hospital Register, Sweden.

## Methods

### Treatment protocol

Preoperatively patients underwent magnetic resonance imaging (MRI) or computed tomography (CT) or both of the primary tumour. An ultrasound or CT-targeted core needle biopsy and fine needle aspiration were taken so that the surgeon was able to excise the biopsy tract during definite tumour surgery. Patients with a high-grade tumour also underwent a CT of the lungs. The primary treatment in all cases was a surgical resection. If the preoperative investigations indicated that adequate surgical margins were not achievable, surgery aimed at marginal surgical margins with postoperative radiation therapy. The treatment protocol recommended, following intralesional surgery, a reoperation when feasible.

Follow-up was recommended for all patients. For high-grade sarcomas, the interval was 2 months during the first 2 years, thereafter three times annually up to 5 years, and for low-grade sarcomas three times annually up to 5 years. Follow-up included a chest X-ray at each visit and an MRI or a CT scan of the primary tumour area 6 months postoperatively and every 6 months up to 2 years and thereafter once annually up to 5 years. In low-grade tumours and synovial sarcomas, an MRI or a CT scan of the primary tumour area together with chest X-ray was recommended after 5 years annually up to 7 years and thereafter once in every 18 months up to 10 years.

### Definition of parameters

Necrosis and vascular invasion were reported as defined by Gustafson *et al* (2003). They were re-evaluated for the present study and classified as absent or present. Tumour size (cm) was recorded as the largest diameter of tumour in the surgical specimen reported by the original pathologist. The pathologist assigned the histological malignancy grade of the tumour based on a four-tiered grading scale modified from Broders *et al* (1939) and Angervall *et al* (1986). Grades 1 and 2 are low grades and 3 and 4 high grades. Subcutaneous tumours with or without cutaneous extension but without involvement of the deep fascia were defined superficial, all others deep.

### Validation material

The southern Sweden STS database is population based and includes adult patients with STS of the extremity or trunk wall. During the 25-year period 1973–1997, 578 patients were included. Patients with metastatic disease at presentation, patients receiving adjuvant chemotherapy and patients with missing data on the assessed and reported parameters were excluded, which left 354 patients for this study. Clinical characteristics of the Helsinki series (test) and the Lund series (validation) are shown in Table 1. In all, 57 patients received postoperative radiation therapy (11 patients after wide surgery, 39 after marginal surgery, and 7 after intralesional surgery). In the validation material, there were more tumours larger than 5 cm. Otherwise, the material was similar to Helsinki material (Table 1).

### Statistical methods

The χ^2^-test and Pearson′s product moment correlation was used to test for associations between factors and the odds ratio to examine the strength of the relationships. Survival curves were calculated according to the Kaplan–Meier method. Sarcoma-specific survival (SSS) was calculated from the date of the diagnosis to death from sarcoma. Deaths due to other causes than sarcoma were censored. Survival curves were compared with the log-rank test. Multivariate survival analyses were performed with the Cox proportional hazards model entering the following covariates: tumour size (⩽10 cm *vs* >10 cm and continuous), necrosis (absent *vs* present), vascular invasion (absent *vs* present), tumour depth (superficial *vs* deep), location (extremity *vs* axis of body), and histological grade (four levels). The covariates were centred around their mean values in the Helsinki data (Table 1). The assumption of proportional hazards was ascertained with complementary log plots and the assumption of a linear relationship between tumour size and survival was assessed by analysis of Cox *β*-coefficients according to tumour size deciles.

For comparison and as a sensitivity analysis, a competing risk regression analysis was performed (Fine and Gray, 1999). The competing risks model estimates the cumulative incidence function and accounts for events competing with the primary outcome. In the current study, death due to sarcoma is the primary event of interest and the competing events are deaths due to other causes.

Based on the fitted Cox regression models 10-year sarcoma-specific survival is estimated for each patient individually. With the *β*-coefficients of the Cox models, a prognostic index (PI) is calculated. The survival curve corresponding to the average PI value is computed, which is similar to the average survival for the total group. If the average PI value is taken as baseline reference, the relative risk (RR) of an individual patient is given by 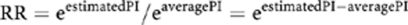


With the average survival curve corresponding to the average PI value and the patient's relative risk, an expected survival curve for a new patient can be computed. For example, if the average cumulative 10-year survival is 0.70, then expected 10-year survival for a patient with the relative risk of RR is 0.70^RR^.

Models were statistically compared according to accuracy of 10-year sarcoma-specific survival prediction in the Helsinki series and in the external Lund validation series. The accuracy was evaluated with regard to discrimination (the area under the ROC curve-AUC), Harrell's *c*, and calibration. The AUC can be interpreted as the probability that for given two subjects, one who will develop an event (sarcoma-specific death) and the other who will not, the model will assign a higher survival probability to the latter. Harrell's *c* estimates the probability that of two randomly chosen patients, the patient with a higher predicted survival will outlive the patient with a lower predicted survival (Harrell *et al*, 1982). An AUC or *c* index of 0.5 indicates a random predictor and 1.0 a perfect predictor. In the AUC analysis, patients who died of intercurrent causes within 10 years are considered sarcoma survivors. In Harrell's *c* patient pairs in which the shorter survival time is censored are discarded from analysis. Calibration was analysed by ranking the patients according to ascending predicted 10-year survival dividing the cohort into ten equally sized groups and calculating the observed 10-year sarcoma-specific survival in each group according to Kaplan–Meier estimates. In addition, the net reclassification improvement (NRI) and the integrated discrimination improvement (IDI) was calculated in the comparison of the previously described model (SIN) and the new proposed model (SAM) with cutoff chosen at estimated survival tertiles (Pencina *et al*, 2008).

All *P*-values are two-sided and *P*-values less than 0.05 are considered statistically significant. A Bonferroni correction is applied in case of multiple comparisons. Statistical analyses were performed using STATA software (version 12.0 StataCorp., College Station, TX, USA). We received approval for this study from the Ministry of Social and Health Affairs and from the Helsinki University Central Hospital Ethics Committee.

### Online web calculator

The final prognostic model is made available online at http://www.prognomics.org/sam, where the user can enter information on the covariates for a new patient and retrieve a 10-year sarcoma-specific survival estimate as described.

## Results

All patients referred for non-metastatic, primary or locally recurrent STS of the extremities or trunk wall to the Soft Tissue Sarcoma Group between August 1987 and December 2002 are included. Exclusion criteria comprised: extraskeletal osteosarcoma, chondrosarcoma, Ewing/PNET family tumour, angiosarcoma, alveolar soft tissue sarcoma, epitheloid sarcoma, clear cell sarcoma, atypical lipoma/grade I liposarcoma, dermatofibrosarcoma protuberans or preoperative radiation therapy. A total of 15 patients with chemotherapy were also excluded. In all, 38 patients included had a locally recurrent tumour. Sarcoma-specific survival of these patients was similar to patients with primary disease at presentation. Of the 38 patients, 26 were referred after first recurrence, 6 after second recurrence, 2 after third recurrence, and 4 after later local recurrence.

In 84 cases, we were unable to retrieve the original histological slides leaving 294 tumours to analysis. Demographic data for missing cases was similar except for histological subtype (Table 1). The 5-year sarcoma-specific survival rate for these 84 patients was 82% (95% CI 0.75–0.91) and at 10 years 74% (95% CI 0.69–0.78).

**Figure 1:**
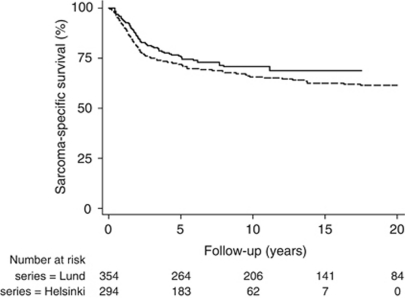
Sarcoma-specific survival in the Helsinki (*n*=294; **—**) and Lund (*n*=354; - - -) series.

Malignant fibrous histiosytoma, liposarcoma, and leiomyosarcoma were the commonest histological subtypes (Table 1). There were no treatment-related deaths. The median follow-up for the patients alive at the end of follow-up was 7.2 years (range 0.3–17.5 years). The 5-year sarcoma-specific survival rate was 75% (95% CI 0.70–0.80) and at 10 years 71% (95% CI 0.64–0.76) (Figure 1).

In four patients, the tumour was a post-irradiation sarcoma. In all, 16 (8%) out of 205 patients with tumour of the extremity were treated with an amputation. A total of 134 patients received postoperative radiation therapy (9 patients after wide surgery, 103 after marginal, and 22 after intralesional surgery).

### Association between tumour characteristics

In pairwise correlation analyses between tumour necrosis, histological grade, vascular invasion, tumour depth, and size statistically significant associations were detected between all variables except for between vascular invasion and tumour depth (Table 2). Tumour location was not associated with any of these factors.

### Univariate survival analysis

In univariate survival analysis, tumour size, necrosis, vascular invasion, histological grade (grade 1 tumours excluded from the analysis due to zero events), and tumour depth (superficial *vs* deep) were significantly associated with sarcoma-specific survival whereas location (axial *vs* extremity) was not (Table 3).

### Multivariate survival analysis and proposed prognostic model

In the multivariate Cox proportional hazards regression tumour size per cm, histological grade per grade, and tumour depth were significant predictors of sarcoma-specific survival. Furthermore, location became significant whereas necrosis and vascular invasion were no longer statistically significant (Table 3). As both of the last-mentioned factors showed relatively large effect sizes (hazard ratios) and have been validated in several previous studied, also these factors were included in the final prognostic model. The relationship between tumour size and survival was assessed by analysis of Cox *β*-coefficients according to tumour size deciles and found to be linear up to a tumour diameter of 15 cm. In patients with a tumour diameter larger than 15 cm, a slight deviation from linearity was seen, but the number of patients in this category was low (*n*=20).

We also performed the competing risks regression in addition to the Cox model for the six parameters in our model. Subhazard ratios were similar to hazard ratios obtained in the Cox model (subhazard ratio; *P*-value): size per cm (1.10; 0.000), necrosis (1.47; 0.224), vascular invasion (1.70; 0.066), grade (1.58; 0.003), depth (3.43; 0.002) and location (1.69; 0.029). We therefore decided to use the Cox regression coefficients for the proposed prognostic model.

**Figure 2:**
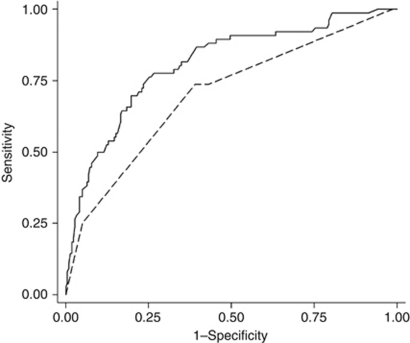
Comparison of prognostic accuracy (area under the ROC curve) between the SIN (- -) (AUC=0.74) and the SAM (**—**) (AUC=0.81) models in prediction of 10-year sarcoma-specific survival in the study series (Helsinki).

When tested on the Helsinki series the proposed model (SAM) based on the Cox regression multivariate model had an AUC of 0.81 (95% CI 0.75–0.87) and concordance index (Harrell's *c*) of 0.79, compared with the SIN model with an AUC of 0.74 (95% CI 0.67–0.81; *P*=0.0007) and concordance index of 0.74, in prediction of sarcoma-specific survival (Figure 2).

### Validation of the prognostic model using an external, independent patient series

**Figure 3:**
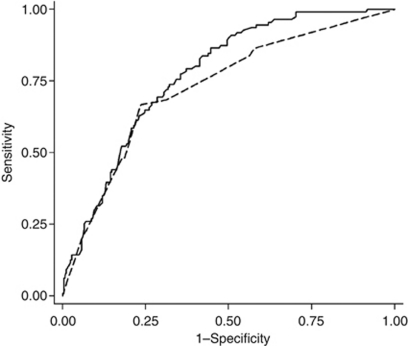
Comparison of prognostic accuracy (area under the ROC curve) between the SIN (- -) (AUC=0.73) and the SAM (**—**) (AUC=0.77) models in prediction of 10-year sarcoma-specific survival in the external validation series (Lund).

**Figure 4:**
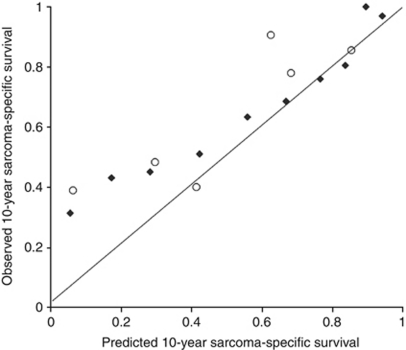
Calibration curve for predicted *vs* observed 10-year sarcoma-specific survival in 10 equally sized groups according to the proposed SAM model (♦) and according to the original SIN model (○) in the external validation series (Lund).

The discrimination according to the AUC of the proposed model was significantly higher also in the validation series. AUC of 0.77 (95% CI 0.72–0.82) and concordance index of 0.77 were detected for SAM model, and 0.73 (95% CI 0.67–0.78; *P*=0.035) and concordance index of 0.73 for SIN model (Figure 3). Calibrations of the proposed SAM model and the original SIN model were analysed in the validation series by plotting the predicted 10-year sarcoma-specific survival against observed survival (Kaplan–Meier estimates) in 10 equally sized groups according to ascending survival (Figure 4). A good concordance is seen in the groups with a predicted 10-year survival of over 50%, whereas a slight underestimation is observed in the groups predicted to have the lowest survival.

Table 4 shows survival reclassification results for the SIN model *vs* the proposed SAM model in the validation series. When the patients were classified into three categories (cutoff at tertiles) on the basis of their predicted 10-year sarcoma-specific survival, the net reclassification improvement (NRI 0.12, *P*=0.03) is significant as well as the integrated discrimination improvement (IDI 0.03, *P*=0.0003). For example, 16% (11 out of 68) of patients who died of sarcoma were reclassified according to the SAM model from the moderate (40–80%) to the more correct poor survival (<40%) group. Correspondingly, 28% (31 out of 112) of 10-year survivors were reclassified from the moderate survival (40–80%) to the more correct favourable survival group (⩾80% sarcoma-specific 10-year survival) (Table 4).

## Discussion

Prognostic tools are valuable when considering patient selection to receive treatments with only limited potential and possible serious side effects. This is especially true for chemotherapy in STS (Pervaiz *et al*, 2008). The largest randomised study failed to show a survival advantage for adjuvant chemotherapy among patients with high-grade STS (Woll *et al*, 2007). Because of major treatment protocol deviations, the effect of adjuvant chemotherapy is still somewhat controversial.

In the present study, we used the SIN system by Gustafson *et al* (2003) as a base and refined it to better discriminate among patients. The original system with dichotomized size, vascular invasion, and necrosis divided patients only to high-risk and low-risk groups. Addition of tumour depth, histological grade, and location together with tumour size now as a continuous variable to a Cox proportional hazards regression-based model forms our new model to predict 10-year sarcoma-specific survival. The two models were statistically compared for accuracy with AUCs of 0.81 and 0.74, *P*=0.0007 and concordance indeces of 0.79 and 0.74, in favour of the new proposed model. We also performed an external independent validation of the model with material from the southern Sweden soft tissue sarcoma database at Lund University Central Hospital. The new proposed model was better to discriminate among patients also in the external validation with AUCs of 0.77 and 0.73, *P*=0.03 and concordance indeces of 0.77 and 0.73. The validation series was older with some differences in the distribution of tumour characteristics and the fact that our model worked well also in the validation series is further proof that the proposed model is valid.

Concordance index with regard to prognostic accuracy was 0.79 for the proposed model. Kattan *et al* (2002) published concordance index of 0.77 for their prognostic model. In external validation of the Kattan model, a concordance index of 0.76 was reported in two separate studies (Mariani *et al*, 2005; Eilber and Kattan, 2007), a concordance index of 0.77 was present in our validation series. Calibration of the model, that is, how close the predicted survival is to the observed, was reported in one of the Kattan model validation studies (Eilber and Kattan, 2007). Results were similar to ours with a tendency towards underestimation in groups with lower predicted survival.

Kattan *et al* (2002) used dichotomised histological grade namely low-grade and high-grade. Their model was adapted to use a three-grade system in one of the validation studies (Mariani *et al*, 2005). Our model uses a four-grade system that is widely used in Scandinavia. We used size on a continuous scale instead of three groups as used by Kattan *et al* (2002). We included also locally recurrent sarcomas, because there is some evidence that prognosis of patients presented with an isolated local recurrence can be comparable to patients presented with primary tumour (Midis *et al*, 1998; Ramanathan *et al*, 2001; Zagars *et al*, 2003). This was true also in our series. As Helsinki is the largest tertiary referral centre, many patients are referred for local recurrence after inadequate local treatment for the primary tumour in district hospitals. Exclusion of considerable amount of patients with locally recurrent tumour at presentation would distort our material.

Our model only includes sarcomas of the limbs and the trunk wall unlike Kattan's. We did not include age because poor physical condition frequently seen among older patients prevents the use of adjuvant therapy. Further, benefit of adjuvant chemotherapy among older patients is less when evaluated by life-years saved. In some prognostic instruments, the tumour histology has also been included as a factor. We chose not to include this in the present study because most histological groups were too small to yield a reliable estimate of its prognostic impact. This will be the subject of further studies in larger patient series. Further, we excluded rare histological subtypes with different biological potential and some with targeted therapy. Histology was not of prognostic value for sarcoma-specific survival in one of the largest series published (Lewis *et al*, 1997).

In a recent study, peripheral tumour growth pattern (infiltrating *vs* pushing growth) was added to the SIN model in addition to size, necrosis, and vascular invasion (Carneiro *et al*, 2011). The authors report improved classification in comparison with the SIN model but unfortunately AUC values are not reported, which complicates comparisons with the current study. Moreover, growth pattern analysis was done on histological macrosections, which are not readily available at all institutions.

Although Helsinki University Central Hospital is the largest sarcoma referral centre in Finland, the number of new STS patients treated annually for sarcoma in the limb or the trunk wall is approximately 50, which limits the number of evaluable patients in comparison with the other sarcoma prognostic tool series. We have treated patients according to the same prospective protocol since 1987 making this series very uniform. Patients with adjuvant chemotherapy were excluded from the analysis. However, these were relatively few (15) in the study series and the exclusion therefore is unlikely to have influenced our model to a greater extent. As these patients would have been assigned a low survival probability by the model, it could explain part of the less optimal calibration detected in the low survival groups. One weakness in our material is that 84 samples were unavailable for re-evaluation. This subgroup was however similar to the group included except for histological subtype: leiomyosarcomas were overrepresented in missing specimen.

Adjuvant chemotherapy has according to the latest meta-analysis a statistically significant but only a moderate effect on survival (Pervaiz *et al*, 2008). Treatment preferred by most institutions, that is, doxorubicin-based combination therapy, is relatively toxic. Most STS patients are elderly and at considerable risk of developing life-threatening or even fatal side effects such as neutropenic infections. Therefore, balancing the benefits of adjuvant chemotherapy against the risks favours withholding adjuvant chemotherapy in those at low risk of dying of their disease. For the purpose of identifying patients with a more favourable survival, the proposed model seems reasonably accurate and well calibrated.

In conclusion, we have created a new prognostic model to estimate survival probability in patients with the commonest subtypes of STS. An external validation was performed showing a good prognostic accuracy and an improvement in accuracy compared with a model with size, necrosis, and vascular invasion only. Our model can be seen as a working formulation to be refined by validation in further external validation studies and is made available online.

## Figures and Tables

**Table 1:** Characteristics of the patients and tumours in the Helsinki and Lund material

**Characteristic**	**Helsinki *N* (%)**	**Helsinki, missing slides (*n*=84) *N* (%)**	**Lund *N* (%)**
*Age (in years)*			
Mean (range)	57 (16–92)	58 (18–92)	63 (17–96)
			
*Sex*			
Male	153 (52)	42 (50)	197 (56)
Female	141 (48)	42 (50)	157 (44)
			
*Tumour size (cm)*			
⩽5	153 (52)	48 (57)	135 (38)
>5 and ⩽10	93 (31)	24 (29)	140 (40)
>10	48 (16)	12 (14)	79 (22)
Median	5	5	6
Median, superficial^a^	3	3	5
Median, deep	7	6	8
			
*Necrosis*			
No	153 (52)	ND	148 (42)
Yes	141 (48)		206 (58)
			
*Vascular invasion*			
No	255 (87)	ND	276 (78)
Yes	39 (13)		78 (22)
			
*Histological grade*			
1	12 (4)	3 (4)	20 (6)
2	71 (24)	23 (27)	54 (15)
3	103 (35)	25 (30)	99 (28)
4	108 (37)	33 (39)	181 (51)
			
*Tumour depth*			
Superficial	109 (37)	28 (33)	117 (33)
Deep	185 (63)	56 (67)	237 (67)
			
*Location*			
Extremity	205 (70)	58 (69)	279 (79)
Axis of body	89 (30)	26 (31)	75 (21)
			
*Histological subtype*			
MFH	123 (42)	20 (24)	181 (51)
LMS	42 (14)	23 (27)	52 (15)
LS	44 (15)	18 (21)	41 (12)
SS	32 (11)	7 (8)	21 (6)
MPNST	13 (4)	0	10 (3)
Others	40 (14)	16 (20)	49 (14)
			
*Surgical margin*			
Intralesional	27 (9)	8 (10)	31 (9)
Marginal	139 (47)	39 (46)	133 (38)
Wide	128 (44)	37 (44)	190 (53)

Abbreviations: MFH=malignant fibrous histiosytoma; LMS=leiomyosarcoma; LS=liposarcoma; SS=synovial sarcoma; MPNST=malignant peripheral nerve sheath tumour; ND=no data.

a Subcutaneous tumours with or without cutaneous extention but without involvement of the deep fascia. Otherwise deep.

**Table 2:** Pairwise correlation between tumour characteristics (Pearson's product moment correlation, Bonferroni correction)

**Characteristic**	**Tumour size**	**Necrosis**	**Vascular invasion**	**Histological grade**	**Tumour depth**	**Location**
Tumour size	1					
Necrosis	0.41^***^	1				
Vascular invasion	0.16^**^	0.29^***^	1			
Histological grade	0.23^***^	0.55^***^	0.30^***^	1		
Tumour depth	0.36^***^	0.18^***^	0.11	0.14^*^	1	
Location	−0.02	0.00	0.03	−0.05	−0.05	1

^***^P<0.0001, ^**^ *P*<0.001, ^*^ *P*<0.01.

**Table 3:** Univariate and multivariate Cox proportional hazards regression of tumour characteristics for sarcoma-specific survival

		**Univariate**		**Multivariate**	
**Characteristic**	***N* (%)**	**HR (95% CI)**	***P***	**HR (95% CI)**	***P***
*Tumour size (cm)*					
⩽5	153 (52)	1		1.10 (1.05–1.15)^a^	<0.0001
5–10	93 (32)	3.09 (1.73–5.52)	<0.0001		
>10	48 (16)	8.24 (4.60–14.75)	<0.0001		
					
*Necrosis*					
No	153 (52)	1		1	
Yes	141 (48)	3.86 (2.34–6.39)	<0.0001	1.60 (0.88–2.90)	0.12
					
*Vascular invasion*					
No	255 (87)	1		1	
Yes	39 (13)	2.80 (1.67–4.70)	<0.0001	1.60 (0.93–2.75)	0.09
					
*Histological grade*					
1	12 (4)	NA^b^		1.57 (1.11–2.22)^c^	0.01
2	71 (24)	1			
3	103 (35)	3.10 (1.36–7.09)	0.007		
4	108 (37)	5.16 (2.31–11.52)	<0.0001		
					
*Tumour depth*					
Superficial	109 (37)	1		1	
Deep	185 (63)	5.54 (2.76–11.11)	<0.0001	3.51(1.71–7.38)	0.001
					
*Location*					
Extremity	202 (69)	1		1	
Axis of body	92 (31)	1.13 (0.70–1.83)	0.61	1.65 (1.01–2.68)	0.04

Abbreviations: HR=hazard ratio; CI=confidence interval; NA=not available.

a per centimetre.

b grade 1 tumours excluded from the analysis due to zero events.

c per grade.

**Table 4:** Reclassification of patients in the validation series (*n*=354) on the basis of 10-year predicted sarcoma-specific survival

	**SAM model**			
	**⩾80%**	**40–80%**	**<40%**	**Total**
*Cases, by predicted survival* a				
⩾80%	10	6		16
40–80%	2	55	11	68
<40%		9	18	27
Total	12	70	29	111
				
*Non-cases, by predicted survival* a				
⩾80%	105	10		115
40–80%	31	75	6	112
<40%		2	14	16
Total	136	87	20	243

Abbreviation: SAM=the proposed extended model.

a 10-year predicted sarcoma-specific survival on the basis of SIN (size, invasion, necrosis) model prognostic factors only.
